# Molecular Characteristics and Promoter Analysis of Porcine *COL1A1*

**DOI:** 10.3390/genes13111971

**Published:** 2022-10-28

**Authors:** Guangming Xiang, Lei Huang, Xiuling Zhang, Nan Wang, Hui Wang, Yulian Mu, Kui Li, Zhiguo Liu

**Affiliations:** 1 Key Laboratory of Animal Genetics Breeding and Reproduction of Ministry of Agriculture and Rural Affairs of China, Institute of Animal Sciences, Chinese Academy of Agricultural Sciences, Beijing 100193, China; 2 Genome Analysis Laboratory of the Ministry of Agriculture and Rural Affairs, Agricultural Genomics Institute at Shenzhen, Chinese Academy of Agricultural Sciences, Shenzhen 518124, China

**Keywords:** *COL1A1*, core promoter, luciferase assay, protein structure and function, pig

## Abstract

*COL1A1* encodes the type I collagen α1 chain, which shows the highest abundance among members of the collagen family and is widely expressed in different mammalian cells and tissues. However, its molecular characteristics are not completely elucidated. In this study, the molecular profiles of *COL1A1* and characteristics of the COL1A1 protein were investigated using a promoter activity assay and multiple bioinformatics tools. The results showed that the 5′ flanking region of porcine *COL1A1* contained two CpG islands, five core promoter sequences, and twenty-six transcription factor-binding sites. In the luciferase assay, the upstream 294 bp region of the initiation codon of *COL1A1* showed the highest activity, confirming that this section is the core region of the porcine *COL1A1* promoter. Bioinformatic analysis revealed that COL1A1 is a negatively charged, hydrophilic secreted protein. It does not contain a transmembrane domain and is highly conserved in humans, mice, sheep, and pigs. Protein interaction analysis demonstrated that the interaction coefficient of COL1A1 with COL1A2, COL3A1, ITGB1, and ITGA2 was greater than 0.9, suggesting that this protein plays a crucial role in collagen structure formation and cell adhesion. These results provide a theoretical basis for further investigation of the functions of porcine *COL1A1*.

## 1. Introduction

The extracellular matrix (ECM) consists of approximately 300 proteins that collectively provide mechanical support and perform signaling functions in mammalian cells [1]. The evolution of these ECM proteins is crucial for the transition to multicellularity, arrangement of cells into functional tissues, and formation of new structures during vertebrate evolution [2]. Collagen is a structural protein in the ECM and is mainly composed of ribbon fibers [3]. Fibril-forming collagens are synthesized as procollagens, which contain a rod-like central triple-helical region with both N- and C-terminal propeptide extensions [4]. Each collagen molecule is a trimer, with three identical peptides forming a homotrimer and at least one different peptide forming a heterotrimer [1]. Collagen is a major building block in many tissues and plays substantial roles in cell proliferation, migration, and differentiation [3].

Type I collagen is the most widely expressed member of the collagen family of proteins and is the main component of the skin, bone, cornea, and other tissues [1]. Type I collagen functions as a tissue support to maintain the integrity of tissues and organs and ensure their normal functioning [2]. The majority of type I collagen exists as heterotrimers consisting of two α1 chains and one α2 chain [5]. A small number of collagen I homotrimers (three molecules of α1 chains) are present in adult skin and embryonic tissues [6,7], and this reduces the efficiency of self-assembling fibrils [8] and increases resistance to proteases [9]. Increased quantities of collagen I homotrimers are closely associated with various cancers and osteogenic dysplasia [1].

*COL1A1* encodes the type I collagen α1 chain, which is associated with cell proliferation, invasion [10,11], and fibrosis [12,13,14]. It is also associated with vascular diseases [15], skeletal injury dysplasia [16], osteogenesis imperfecta [17], osteoporosis [18], and other pathological conditions [19]. In addition, the expression of this gene is significantly upregulated in various cancers such as liver, pancreatic, breast, and ovarian cancers, making it an important biomarker for cancer [20,21,22,23]. Porcine *COL1A1* is located on chromosome 12 and has a total length of 18,094 bp (NC_010454) and high GC content (59.8%). According to the gene expression atlas analysis (https://www.ebi.ac.uk/gxa/home, accessed on 10 June 2021), *COL1A1* is widely expressed in different porcine tissues and cells, such as the corpus callosum, stomach, epididymis, omentum, mesenteric lymph nodes, and porcine fetal fibroblasts, suggesting that the *COL1A1* locus can be used as a candidate safe harbor to mediate the wide expression of foreign genes in the porcine genome [24]. However, the transcriptional regulatory mechanisms and structure of porcine *COL1A1* have not been extensively studied. The gene promoter is a complex area containing numerous binding sites for transcription factors, which determines the transcription start site and frequency [25]. Therefore, the recognition of promoters and identification of regulatory mechanisms of transcription is essential. In this study, the promoter of porcine *COL1A1* was predicted and analyzed, and promoter activity was verified. Furthermore, the structure and function of porcine COL1A1 were predicted. The results provide valuable information for further studies on the functions of porcine *COL1A1*.

## 2. Materials and Method

### 2.1. Bioinformatics Analysis of 5′ Flanking Region of Porcine COL1A1 Gene

The 5′ flanking region of *COL1A1* (2405 bp before the initiation codon) was obtained from the National Center for Biotechnology Information database. The core active region of the *COL1A1* promoter was predicted using Network Promoter Prediction online software. CpG islands were predicted using MethPrimer. Putative promoter transcription factor-binding sites were predicted using Jaspar 2022. Sequences 500 bp before the initiation codon in humans, mice, sheep, and pigs were downloaded from the National Center for Biotechnology Information database and compared with the results of analysis of the core promoter of porcine *COL1A1*. The detailed software information is presented in Table 1.

### 2.2. Amplification of Porcine COL1A1 Promoter with Different Lengths

To construct porcine *COL1A1* promoter vectors of different lengths, genomic DNA was extracted from porcine embryonic fibroblast (PEF) cells using the TIANapm Genomic DNA Kit (Tiangen, Beijing, China) and used as a template for polymerase chain reaction (PCR). Five promoters of pig *COL1A1* with different lengths were obtained. As a positive control, the cytomegalovirus (CMV) promoter was also cloned from the pX458 vector using PCR. The promoters included 334 bp (−294–0 bp, using primers P-294-1F and P-1R), 498 bp (−458–0 bp, using primers P-458-1F and P-1R), 905 bp (−865–0 bp, using primers P-865-1F and P-1R), 1478 bp (−1438–0 bp, using primers P-1438-1F and P-1R), 2445 bp (−2405–0 bp, using primers P-2405-1F and P-1R), and 548 bp (−508–0 bp, using primers CMV-F and CMV-R). The primer sequences are shown in Table 2. PCR was performed using a KOD FX high-fidelity enzyme system (Toyobo, Shanghai, China) under the following conditions: 94 °C for 2 min; 36 cycles at 94 °C for 10 s, 60 °C for 30 s, and 68 °C for 1 kb/min (time varied depending on the fragment length); 68 °C for 2 min; and 4 °C holds. All PCR products contained a 40 bp homologous arm sequence for seamless cloning. The PCR products were purified and recovered according to the instructions provided in the Zymoclean^TM^ Gel DNA Recovery Kit (ZYMO RESEARCH, Irvine, CA, USA).

### 2.3. Luciferase Vector Construction

The pig *COL1A1* promoter constructs of different lengths, namely pGL3-294, pGL3-458, pGL3-865, pGL3-1438, pGL3-2405, and the CMV promoter, pGL3-CMV, were ligated into the pGL3-Basic vector using seamless cloning technology [26]. To determine the transfection efficiency, pGL4.75 vector (Promega, Beijing, China) was used as an internal control. For vector construction, the pGL3-Basic vector (Promega, Beijing, China) was linearized by restriction endonuclease HindIII in a reaction mix containing 2 μL plasmid DNA, 1 μL HindIII, 5 μL 10× NEBuffer 2.1, and 42 μL water at 37 °C for 2 h. Recovery was performed using a Zymoclean^TM^ Gel DNA Recovery Kit in accordance with the manufacturer’s protocol. The purified PCR products and linearized vectors were reconstituted and ligated as follows: 1 μL PCR product, 1 μL linearized vector, 5 μL 2× ClonExpress Mix, and water (to a total reaction volume of 10 μL), followed by a reaction at 50 °C for 30 min. The recombinant plasmids were transformed into DH5*α* competent cells and cultivated on Luria-Bertani solid medium containing ampicillin. The next day, a single colony was selected for sequencing using the universal primer, RVPrimer3 (Tsingke, Beijing, China). Colonies with correct sequences were expanded and cultured, and all plasmids were extracted in large quantities using EndoFree Plasmid Midi Kits (Cwbiotech, Jiangsu, China) for subsequent cell transfection.

### 2.4. Cell Culture and Transfection

PEFs were isolated from a single 35-day-old Large White pig fetus by our lab, and maintained in Dulbecco’s modified Eagle’s medium (Gibco, Grand Island, NY, USA) supplemented with 15% fetal bovine serum (Gibco), 1% penicillin/streptomycin (Gibco), 1% GlutaMAX (Gibco), 1% non-essential amino acids (Gibco), and 1% sodium pyruvate (Gibco). The cells were passaged for six generations prior to transfection. Porcine intestinal epithelial (IPI-2I) cells, a cell line established from the porcine ileum, were a kind gift from Prof. Shaobo Xiao (Huazhong Agricultural University, Wuhan, China). The cells were cultured in Dulbecco’s modified Eagle’s medium supplemented with 10% fetal bovine serum, 1% penicillin/streptomycin, and 1% GlutaMAX. All cells were maintained at 37 °C and 5% CO_2_ in a humidified incubator (Forma Series 3111; Thermo Fisher Scientific, Waltham, MA, USA). Both PEF and IPI-2I cells were mycoplasma-free.

The cells were transferred into a 10 cm dish two days before transfection. When the cells reached 80–90% confluence, they (2 × 10^5^) were co-transfected with 2 μg pGL3 series firefly luciferase plasmids and 10 ng *Renilla* luciferase plasmids using a Basic Primary Fibroblasts Nucleofector Kit (Lonza, Basel, Switzerland) in accordance with the manufacturer’s instructions. The optimal transfection procedures for the PEFs and IPI-2I cells were U-023 and S-005, respectively. The cells were then transferred into 12-well plates and cultured at 37 °C in an incubator with 5% CO_2_. The medium was replaced 6 h after transfection.

### 2.5. Luciferase Reporter Gene Assay

Transcriptional activity of the porcine *COL1A1* promoter was detected using a dual-luciferase reporter system (Promega, Beijing, China). Twenty-four hours after transfection, the cells were harvested for lysis, and 20 μL of the supernatant lysate was transferred to a new opaque white 96-well plate. Subsequently, 100 μL of firefly fluorescence substrate was added to each well, and the fluorescence signal intensity of the firefly was detected using a microplate reader (SpectraMax M5, Molecular Devices, San Jose, CA, USA). *Renilla* luciferase substrate (100 μL) was added to detect the *Renilla* luciferase signal, and relative luciferase activity was calculated as the ratio of firefly fluorescence to *Renilla* luciferase fluorescence. Transfection efficiency was based on co-transfected *Renilla* luciferase values. For each construct in each experiment, at least three transfections were carried out.

### 2.6. Structural Analysis of Porcine COL1A1 Protein

Amino acid sequences of COL1A1 from different species, including humans, mice, sheep, and pigs, were downloaded from the UniProt database and compared. The physicochemical properties were analyzed using ProtParam. Hydrophilicity or hydrophobicity was predicted using ProtScale. Signalp-5.0 and TMHMM-2.0 software were used to analyze the signal peptide and transmembrane domain of the protein. The secondary structure, domain, and tertiary structure of COL1A1 protein were predicted using SOPMA, InterPro domain, and Swiss-Model software. Protein interaction analysis was performed using STRING software. Detailed information on the software and websites is provided in Table 1.

### 2.7. Statistical Analysis

The relative luciferase activity (ratio of firefly fluorescence to *Renilla* luciferase fluorescence) was presented as the means ± standard errors (SEM). Differences were accessed and analyzed by using a one-way analysis of variance (ANOVA) followed by Fisher’s least significant difference test as a multiple comparison test with SPSS 20.0 (SPSS, Inc., Chicago, IL, USA). *p* values less than 0.05 were considered significantly different. Data were virtualized using GraphPad Prism 6.0.0 (La Jolla, CA, USA).

## 3. Results

### 3.1. Bioinformatics Analysis of 5′ Flanking Region of Porcine COL1A1

**Figure 1:**
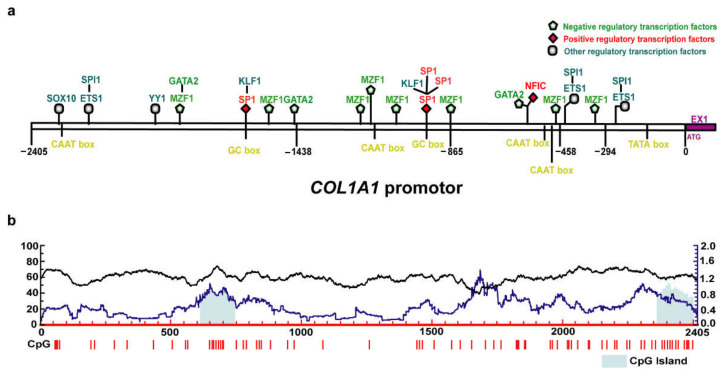
Bioinformatics analysis of 5′ flanking region of porcine *COL1A1*. (**a**) Schematic representation of the transcription factor-binding sites prediction and promotor structure in 5′ flanking region of porcine *COL1A1*; (**b**) CpG islands prediction in 5′ flanking region of porcine *COL1A1*.

To identify the promoter of porcine *COL1A1*, bioinformatics analysis tools were used to analyze the 5′ flanking region of the gene. The results showed that the 5′ flanking region of porcine *COL1A1* had the typical structure of eukaryotic promoters, with one TATA box, two GC-boxes, and four CAAT boxes (Figure 1a). Furthermore, two CpG islands were detected between −1792 to −1661 bp and −143 to −6 bp (Figure 1b). Five core promoter sequences were also detected in this region (Table 3).

Transcription factors in the gene promoter regions determine the transcription frequency and position of the transcription start site. Therefore, we predicted the transcription factor-binding sites in the 5′ flanking region of *COL1A1* using Jaspar, an online analysis software. The results revealed 26 transcription factor-binding sites in the 5′ flanking region of porcine *COL1A1* (Figure 1a, Supplementary Table S1), including transcription factor PU.1 (SPI1), ETS proto-oncogene 1 (ETS1), myeloid zinc finger 1 (MZF1), nuclear factor I C (NFIC), GATA-binding protein 2 (GATA2), specificity protein 1 (SP1), Krüppel-like factor 1 (KLF1), Yin Yang 1 (YY1), and SRY-related HMG-box 10 (SOX10). Among the transcription factor-binding sites, the frequency of MZF1-binding sites was relatively high. Furthermore, according to KnockTF2.0 software and previous studies, the transcription factors MZF1 and GATA2 may negatively regulate *COL1A1* expression [27,28,29]; the transcription factors SP1 and NFIC may positively regulate *COL1A1* expression [30,31]; and other transcription factors, including YY1 [32], ETS1, SPI1 [33,34], SOX10 [35,36], and KLF1 [37,38], may not significantly affect *COL1A1* expression (Figure 1a).

**Figure 2:**
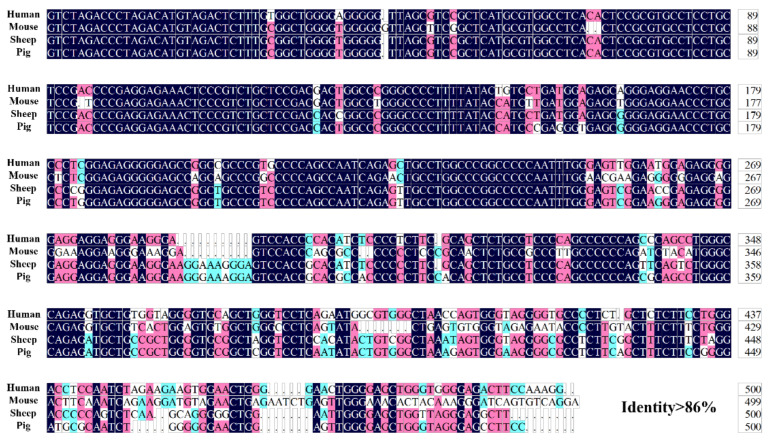
Sequence alignment of 500 bp before the initiation codon of *COL1A1* in human, mouse, sheep, and pig.

In addition, it has been reported that the core promoter of *COL1A1* is located on the 500 bp sequence before the initiation codon in mice and humans [39,40]. The 500 bp sequences before the initiation codon of *COL1A1* were compared in humans, mice, sheep, and pigs, we found that these sequences were highly conserved with greater than 86% identity (Figure 2). Furthermore, two promoter sequences and one CpG island were predicted in this region, indicating that the core promoter of porcine *COL1A1* is 500 bp upstream of the initiation codon.

### 3.2. Analysis of Transcriptional Activity of Porcine COL1A1 Promoter by Luciferase Reporters

**Figure 3:**
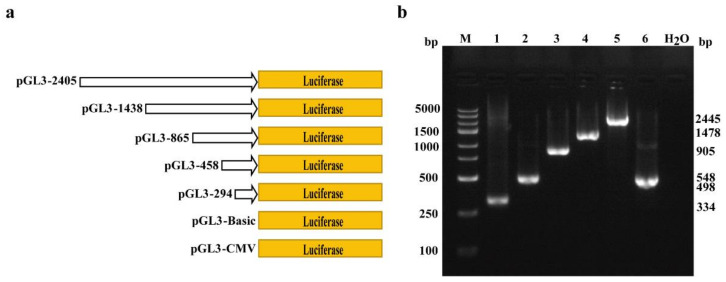
Construction of luciferase reporter vectors with different lengths of porcine *COL1A1* promoter. (**a**) Schematic diagram of the luciferase vectors with different lengths of porcine *COL1A1* promoters (294–2405 bp) and control vectors (pGL3-CMV and pGL3-Basic); (**b**) M, DL250 maker; lane 1, 2, 3, 4, 5, and 6 represent the PCR products of insert fragments in the pGL3-294, pGL3-458, pGL3-865, pGL3-1438, pGL3-2405, and pGL3–CMV vectors, respectively.

**Figure 4:**
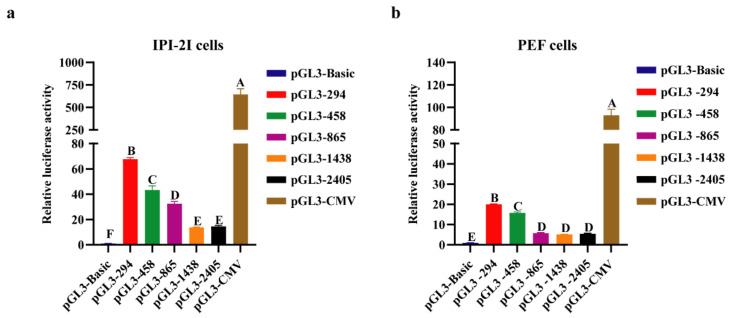
Transcriptional activity of porcine *COL1A1* promoter. Luciferase reporter plasmids containing different lengths of porcine *COL1A1* promoter (294–2405 bp) or control vectors (pGL3-Basic and pGL3-CMV) were transiently transfected into IPI-2I and PEF cells, respectively. Transfection efficiency was normalized by co-transfection with *Renilla* luciferase vector. For each vector in each experiment, three transfections were carried out. The relative luciferase activity is presented as the SEM. Differences between multiple groups were accessed by ANOVA with SPSS 20.0. Groups with different capital letters (A-F) are significantly different (*p* < 0.05). (**a**) Activity analysis of the porcine *COL1A1* promoter of different lengths in IPI-2I cells; (**b**) Activity analysis of the porcine *COL1A1* promoter of different lengths in PEF cells.

To determine the core promoter sequence, pGL3 vectors with different lengths of porcine *COL1A1* promoter were constructed (Figure 3), and the promoter activity was detected using a dual-luciferase reporter system. The results showed that the luciferase activity of the pGL3-294 (−294–0 bp) vector was significantly higher (*p* < 0.05) than that of the pGL3-458 (−458–0 bp), pGL3-865 (−865–0 bp), pGL3-1438 (−1438–0 bp), and pGL3-2405 (−2405–0 bp) vectors (Figure 4). These results indicate that the 294 bp sequence before the initiation codon is the core promoter of porcine *COL1A1.* Furthermore, the activity of the porcine *COL1A1* promoter was similar in IPI-2I and PEF cells.

### 3.3. Amino Acid Sequence Analysis of Porcine COL1A1

**Figure 5:**
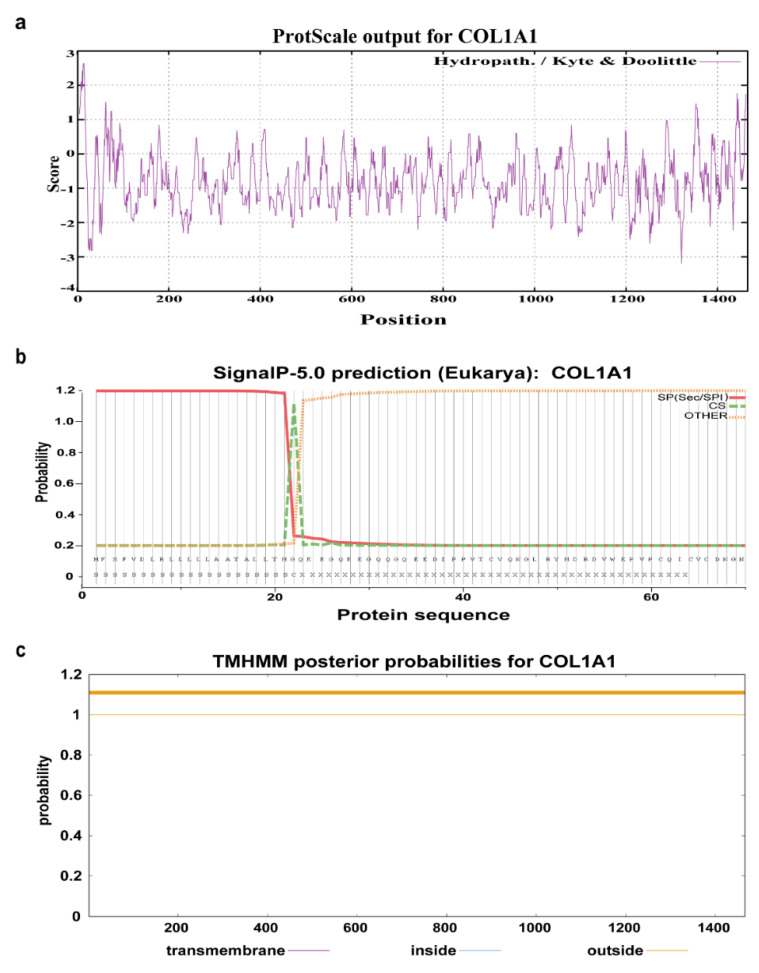
Amino acid sequence analysis of porcine COL1A1. (**a**) Hydrophilicity or hydrophobicity prediction of porcine COL1A1; (**b**) Signal peptide prediction of porcine COL1A1; (**c**) Transmembrane domain prediction of porcine COL1A1.

The amino acid sequences of the human, mouse, sheep, and pig COL1A1 were downloaded from the UniProt database and compared. The results showed that their sequence similarity was greater than 89% (Supplementary Figure S1), indicating that COL1A1 is highly conserved and plays similar roles in these organisms. Physical and chemical property analysis showed that COL1A1 is composed of 1466 amino acids, with a molecular size of 139 KDa and a theoretical isoelectric point of 5.60. The protein is acidic and contains 127 positively charged amino acid residues (Arg and Lys) and 140 negatively charged amino acid residues (Asp and Glu). The most abundant amino acid is Gly (26.5%) and the minor amino acid is Trp (0.4%). The predicted instability coefficient was 32.31 and lipid solubility coefficient was 38.27, indicating that COL1A1 is a stable protein. The average hydrophilic coefficient of COL1A1 was −0.794, and the predicted values for most amino acids were negative, indicating that the protein is hydrophilic (Table 4 and Figure 5a). Signalp-5.0 software was used to predict the signal peptide cleavage sites of porcine COL1A1. The results revealed restriction sites at sites 22 and 23 (Figure 5b), indicating that amino acids 1–22 are signal peptide sequences and that the porcine COL1A1 is a secretory protein. Analysis using the UniProt database showed that the protein was mainly present in the ECM and cytoplasm. Furthermore, TMHMM-2.0 was used to predict the transmembrane domain, which showed that all amino acids in COL1A1 were outside of the membrane and that there was no transmembrane domain (Figure 5c), indicating that porcine COL1A1 is a non-transmembrane protein.

### 3.4. Structural and Functional Prediction of Porcine COL1A1

**Figure 6:**
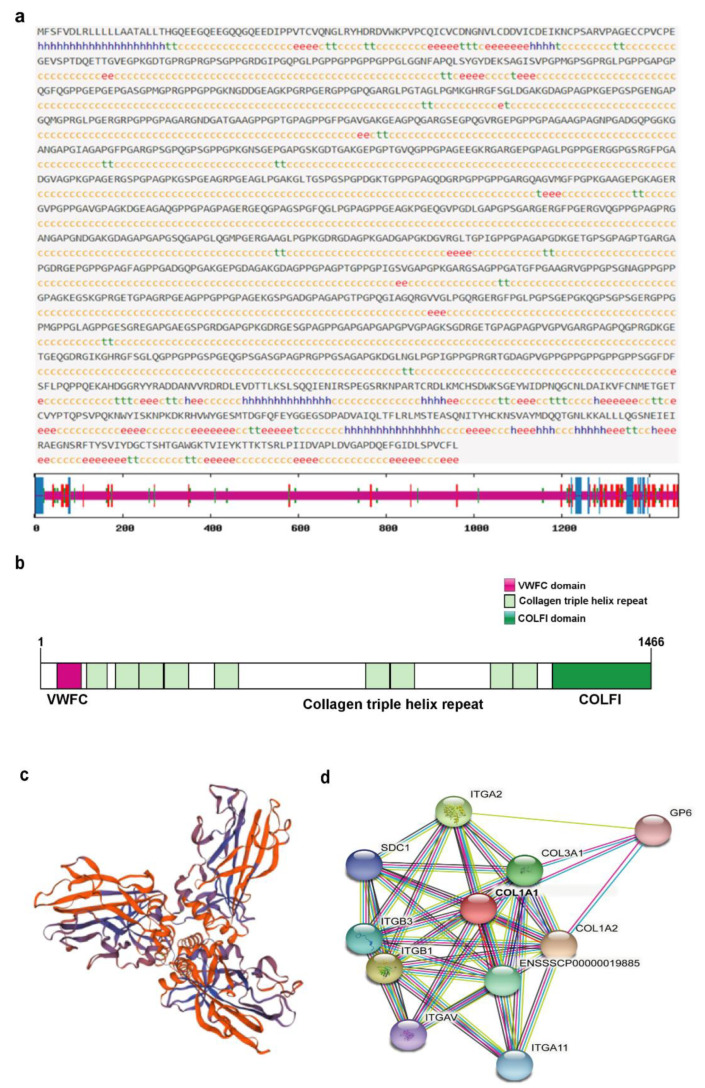
Structural and functional analyses of porcine COL1A1. (**a**) Predicted secondary structure (H and blue line, α helix; t and green line, β turn; e and red line, Extended chain; c and purple line, Random coil); (**b**) Domain prediction; (**c**) Tertiary structure prediction; (**d**) Protein interaction analysis.

Prediction of the secondary structure of porcine COL1A1 showed that α helix, β turn, extended chain, and random coil accounted for 4.71%, 3.96%, 7.98%, and 83.36% of the sequence, respectively (Figure 6a). According to InterPro Domain prediction software, COL1A1 belongs to the type I collagen family and contains a Von Willebrand factor type C (VWFC) domain, fibrillar collagens C-terminal (COLFI) domain, and multiple collagen triple helix repeats (Figure 6b). The tertiary structure of porcine COL1A1 was predicted online using Swiss-Model software. The tertiary structure was composed of the human collagen A1 chain (5K31.1.a) as a template with a sequence consistency of greater than 95%. The overall structure of porcine COL1A1 (trimer) was a flower, with a stalk, base, and three petals [1] (Figure 6c). Interactions between porcine COL1A1 and other proteins were analyzed online using STRING software. The results showed that the interaction coefficients of COL1A1 with COL1A2, COL3A1, ITGB1, and ITGA2 were greater than 0.9. COL1A1 also interacted with ITGB3, ITGA11, SDC1, ITGAV, and GP6 with interaction coefficients greater than 0.8 (Figure 6d).

## 4. Discussion

Porcine COL1A1, a hydrophilic and secretory protein, is one of the main proteins of the ECM and an important component of the skin, bone, tendon, and various other tissues. It provides tissue support and has a wide range of biological functions. Database analysis showed that *COL1A1* is widely expressed in different pig tissues, and highly expressed in the corpus callosum, stomach, epididymis, omentum, mesenteric lymph nodes, and skeletal muscle. However, reports on the transcriptional regulatory mechanisms and structure of porcine *COL1A1* are limited. Promoter recognition and identification are crucial for the transcriptional regulation of genes [41]. Therefore, we analyzed the promoter characteristics of porcine *COL1A1*, identified its core promoter region, and found that several transcription factors may have substantial roles in regulating the expression of *COL1A1*. Further analysis of its protein structure and interaction is valuable for determining the transcriptional regulation mechanism and function of porcine *COL1A1*.

Bioinformatics analysis and luciferase assays are common methods for studying the core and regulatory regions of promoters [42,43]. To understand the transcriptional regulatory mechanism of porcine *COL1A1*, we analyzed the sequence characteristics of its 5′ flanking region. The 5′ flanking region sequence of the porcine *COL1A1* promoter contained the typical CpG islands, TATA boxes, CAAT boxes, and multiple transcription factor-binding sites of eukaryotic promoters. Sequence alignment analysis revealed that the 500 bp sequences before the initiation codon (ATG) in humans, mice, sheep, and pigs were highly conserved. In addition, studies in humans and mice have shown that the core promoter of *COL1A1* is in this region [30,44]. Considering that this region contains one CpG island, a TATA box, a CAAT box, and two predicted promoter sequences, as previously described, the core promoter of porcine *COL1A1* may be located 500 bp before the ATG codon. The results of the luciferase assay in porcine PEF and IPI-2I cells showed that the core promoter was 294 bp before ATG, although the porcine *COL1A1* promoter (2405 bp before ATG) showed promoter activity.

Interestingly, the activity of the promoter decreased significantly as the promoter fragment increased in length in different porcine cells and showed the lowest level for the promoter of −2405 to −1458 bp. Negative regulatory elements may be present in the region from −1458 to −294 bp. Hence, we predicted the transcription factors in this region and mainly detected MZF1, SP1, GATA2, NFIC, and other transcription factor-binding sites. MZF1, a member of the C2H2-type zinc finger protein family, is involved in the proliferation and differentiation of blood cells [45] and tumorigenicity [46]. MZF1 may act as a negative regulator of porcine *COL1A1* based on comparison with transcription factors that bind at −294 to 0 and −458 to −294 bp. This result is consistent with the findings of previous studies which demonstrated that MZF1 can negatively regulate *COL1A1* expression in gastric adenocarcinoma and breast cancer [27,28]. As a basic transcription factor, SP1 belongs to the C2H2-type zinc finger protein family and regulates the expression of genes related to various processes such as cell growth, apoptosis, differentiation, and the immune response [47]. Many studies have shown that SP1 promotes the transcriptional activation of genes [48,49,50] and that SP1 and COL1A1 are highly expressed in a variety of cancers, suggesting that SP1 and COL1A1 are positively correlated. Li et al. (1995) found that SP1 significantly enhances the promoter activity of *COL1A1* in humans, thus positively regulating its expression [30]. However, Nehls et al. (1991) found that SP1 and NFIC (also known as NF-I) have a common binding site in the promoter region of *COL1A1* in NIH3T3 fibroblasts in mice, and SP1 may interfere with the transcriptional activation of NFIC. However, in the herpes simplex virus thymidine kinase (TK1) promoter, the binding sites of SP1 and NFIC are inconsistent, and both SP1 and NFIC can enhance transcriptional activity [31]. We did not detect a common binding site between SP1 and NF-I in the porcine *COL1A1* promoter, suggesting that both SP1 and NFIC positively regulate the expression of porcine *COL1A1*. GATA2 is a member of the GATA family of transcription factors, which have important roles in cell proliferation and differentiation. A previous study reported a negative correlation between GATA2 and COL1A1 expression in patients with breast and ovarian cancers [23]. Moreover, *GATA2*-knockdown in vascular endothelial cells led to upregulated expression of *COL1A1* [29], suggesting that GATA2 also negatively regulates *COL1A1*. In addition, the CEO database showed that the GlI–Krüppel family of zinc finger protein transcription factors (YY1) [32], tryptophan aggregation factor family (ETS1 and SPI1) [33,34], *SOX* gene family (SOX10) [35,36], and KLF family (KLF1) [37,38] may have no significant effect on *COL1A1* expression. The effect of the overexpression or knockdown of related transcription factors on the activity or expression of the porcine *COL1A1* promoter should be further explored. In addition, the detection of GpC islands in the promoter region suggests that DNA methylation plays an important role in regulating *COL1A1* expression.

Protein domains are units of protein structure, function, and evolution [51]. Porcine COL1A1 belongs to the type I collagen family and contains VWFC, multiple collagen triple helix repeats, and COLFI domain. The VWFC domain exists in multidomain or multifunctional proteins involved in maintaining homeostasis and is related to the formation of complex protein structures [52,53]. Proteins containing VWFC domains are involved in various biological processes, such as cell adhesion, migration, and signal transduction. The sequence of the triple helix repeat domain predominantly contains repeats of the glycine–X–Y motif, where X and Y can be any residue but are frequently proline and hydroxyproline. This domain can be post-translationally modified by proline hydroxylase to form hydroxyproline residues, which are associated with scurvy and the host immune defense [54,55,56]. There are many globular proteins between the triple helix repeats that can bind to various substrates. One of these domains is at the C-terminus of fibrotic collagen in the COLFI domain. The C-terminal precursor peptide of precursor collagen controls the intracellular assembly of procollagen molecules and extracellular assembly of collagen fibrils. Particularly, in the presence of different types of collagen in cells, the COLFI domain determines the connection between different peptide chains and plays a crucial role in tissue growth and repair [3,19]. Analysis of protein interactions can also provide insight into the functions of proteins in different biological processes. In this study, COL1A1 combined with COL1A2 and COL3A1 to form precursor collagen, indicating that COL1A1 is a component of various collagens. It may also bind to integrin receptors ITGB1 and ITGA2 for cell adhesion and indirectly interact with actin to link the extracellular matrix to the intracellular skeletal network.

## 5. Conclusions

The 5′ flanking region of porcine *COL1A1* exhibits typical eukaryotic promoter characteristics, with five promoter sequences, two CpG islands, and multiple negative transcription factor binding sites that regulate its expression. The core promoter region is 294 bp upstream of the initiation codon. As a negatively charged, hydrophilic secreted, and non-transmembrane protein, COL1A1 may play a crucial role in collagen structure formation and cell adhesion. This study provides an important theoretical basis for further studies of the transcriptional regulatory mechanism and functions of porcine *COL1A1*.

## Figures and Tables

**Table 1:** Bioinformatics analysis software.

Software/Database	Website
MethPrimer	http://urogene.org/cgi-bin/methprimer2/MethPrimer.cgi, accessed on 15 July 2022
Network Promoter Prediction	https://fruitfly.org/seq_tools/promoter.html, accessed on 15 July 2022
KnockTF2.0	http://www.licpathway.net/KnockTFv2/index.php, accessed on 15 July 2022
Jaspar 2022	https://jaspar.genereg.net/, accessed on 15 July 2022
UniProt	https://www.uniprot.org/, accessed on 20 July 2022
ProtParam	https://web.expasy.org/protparam/, accessed on 20 July 2022
ProtScale	https://web.expasy.org/protscale/, accessed on 20 July 2022
SignalP-5.0	https://services.healthtech.dtu.dk/service.php?SignalP-5.0, accessed on 21 July 2022
TMHMM-2.0	https://services.healthtech.dtu.dk/service.php?TMHMM-2.0, accessed on 21 July 2022
SOPMA	https://npsa-prabi.ibcp.fr/cgi-bin/npsa_automat.pl?page=npsa_sopma.html, accessed on 23 July 2022
InterPro domain	https://www.ebi.ac.uk/interpro/, accessed on 23 July 2022
SWISS-MODEL	https://swissmodel.expasy.org/interactive, accessed on 23 July 2022
STRING	https://cn.string-db.org/, accessed on 23 July 2022

**Table 2:** Primer sequences.

Primer	Primer Sequence (5′→3′)
P-2405-1F	*TCGAGATCTGCGATCTAAGT*ATGCTTGGAAACCTTCTGCC
P-1438-1F	*TCGAGATCTGCGATCTAAGT*AGACGGAGGCTAGGGTAGAG
P-865-1F	*TCGAGATCTGCGATCTAAGT*CCCACCCCATCTCTCTCAAT
P-458-1F	*TCGAGATCTGCGATCTAAGT*ATTGCGCATCCCCGGAAGAA
P-294-1F	*TCGAGATCTGCGATCTAAGT*CTTTCCCTTCCCTTCCCTCC
P-1R	*ACCAACAGTACCGGAATGCC*GTCTAGACCCTAGACATGTAGACT
CMV-F	*TCGAGATCTGCGATCTAAGT*CGTTACATAACTTACGGTAAATG
CMV-R	*ACCAACAGTACCGGAATGCC*AGCTCTGCTTATATAGACCT

Note: Italic bases indicate the homologous arm sequence for seamless cloning and bases not in italic indicate the primers for specific amplification of different fragments.

**Table 3:** Predicted promotor sequence in 5′ flanking region of porcine *COL1A1*.

Number	Start (bp)	End (bp)	Score	Promoter Sequence
1	−2069	−2019	0.86	AGACTGCCACTCTAAAGGGGTGCCCACCACCCTGAGGGTCAGGTCCCTGG
2	−1247	−1197	0.87	GGAAACATCCTTTAAAAGAAGGACACTCACCTGCAATTCCATTTTGAACT
3	−783	−733	0.86	ACAGCCTCAAAATAAAAATCCTCTCATCCGCACCCACCCCCAAATATCTG
4	−409	−359	0.92	AGCCCACAGTATATTGAGGACCGAGCCGCACCCCAGCGGCAGCATCTCTG
5	−157	−107	1	CTCGGGATGGTATAAAAGGGGCCCGGGCCAGTGGTCGGAGCAGACGGGAG

**Table 4:** Physicochemical properties of porcine COL1A1.

Category	Amount	Property
Molecular weight	139 kDa	
Theoretical isoelectric point	5.6	Partial acid
Instability index (II)	32.31	Stable protein
Aliphatic index	38.27	
Grand average of hydropathicity	−0.794	Hydrophilic protein
Formula	C_5998_H_9314_N_1838_O_1946_S_31_	
Total number of negatively charged residues (Asp + Glu)	140	
Total number of positively charged residues (Arg + Lys)	127	
The highest or minor amino acid contents	Gly, 26.5%; Trp, 0.4%	

## Data Availability

Not applicable.

## Supplementary Materials

The following supporting information can be downloaded at: https://www.mdpi.com/article/10.3390/genes13111971/s1. Supplementary Figure S1. Amino acid sequence alignment of COL1A1 in humans, mice, sheep, and pigs. Red boxes indicate the signal peptides. Supplementary Table S1. Predicted transcription factor-binding sites in the promoter region of porcine *COL1A1* in Jarspar.

## References

[1] Sharma U., Carrique L., Vadon-Le Goff S., Mariano N., Georges R.N., Delolme F., Koivunen P., Myllyharju J., Moali C., Aghajari N. (2017). Structural basis of homo-and heterotrimerization of collagen I. Nat. Commun..

[2] Hynes R.O., Naba A. (2012). Overview of the matrisome—An inventory of extracellular matrix constituents and functions. Cold Spring Harb. Perspect. Biol..

[3] Ricard-Blum S. (2011). The collagen family. Cold Spring Harb. Perspect. Biol..

[4] Hulmes D.J. (2002). Building collagen molecules, fibrils, and suprafibrillar structures. J. Struct. Biol..

[5] Han S., McBride D.J., Losert W., Leikin S. (2008). Segregation of type I collagen homo-and heterotrimers in fibrils. J. Mol. Biol..

[6] Uitto J. (1979). Collagen polymorphism: Isolation and partial characterization of alpha 1(I)-trimer molecules in normal human skin. Arch. Biochem. Biophys..

[7] Jimenez S.A., Bashey R.I., Benditt M., Yankowski R. (1977). Identification of collagen alpha1(I) trimer in embryonic chick tendons and calvaria. Biochem. Biophys. Res. Commun..

[8] McBride D.J., Kadler K.E., Hojima Y., Prockop D.J. (1992). Self-assembly into fibrils of a homotrimer of type I collagen. Matrix.

[9] McBride D.J., Choe V., Shapiro J.R., Brodsky B. (1997). Altered collagen structure in mouse tail tendon lacking the alpha 2(I) chain. J. Mol. Biol..

[10] Jia R., Wang C. (2020). MiR-29b-3p Reverses Cisplatin Resistance by Targeting COL1A1 in Non-Small-Cell Lung Cancer A549/DDP Cells. Cancer Manag. Res..

[11] Kim K., Kim Y.J. (2022). RhoBTB3 Regulates proliferation and invasion of breast cancer cells via Col1a1. Mol. Cells.

[12] Tao R., Fan X.X., Yu H.J., Ai G., Zhang H.Y., Kong H.Y., Song Q.Q., Huang Y., Huang J.Q., Ning Q. (2018). MicroRNA-29b-3p prevents Schistosoma japonicum-induced liver fibrosis by targeting COL1A1 and COL3A1. J. Cell Biochem..

[13] Nosalski R., Siedlinski M., Denby L., McGinnigle E., Nowak M., Cat A.N.D., Medina-Ruiz L., Cantini M., Skiba D., Wilk G. (2020). T-cell-derived miRNA-214 mediates perivascular fibrosis in hypertension. Circ. Res..

[14] Mavrogeorgis E., Mischak H., Latosinska A., Vlahou A., Schanstra J.P., Siwy J., Jankowski V., Beige J., Jankowski J. (2021). Collagen-derived peptides in CKD: A link to fibrosis. Toxins.

[15] Bou-Gharios G., Ponticos M., Rajkumar V., Abraham D. (2004). Extra-cellular matrix in vascular networks. Cell Prolif..

[16] Robinson M.E., Rauch D., Glorieux F.H., Rauch F. (2022). Pubertal growth in osteogenesis imperfecta caused by pathogenic variants in COL1A1/COL1A2. Genet. Med..

[17] Marini J.C., Forlino A., Bächinger H.P., Bishop N.J., Byers P.H., Paepe A., Fassier F., Fratzl-Zelman N., Kozloff K.M., Krakow D. (2017). Osteogenesis imperfecta. Nat. Rev. Dis. Primers.

[18] Moradifard S., Hoseinbeyki M., Emam M.M., Parchiniparchin F., Ebrahimi-Rad M. (2020). Association of the Sp1 binding site and -1997 promoter variations in COL1A1 with osteoporosis risk: The application of meta-analysis and bioinformatics approaches offers a new perspective for future research. Mutat. Res. Rev. Mutat. Res..

[19] Giunta C., Chambaz C., Pedemonte M., Scapolan S., Steinmann B. (2008). The arthrochalasia type of Ehlers-Danlos syndrome (EDS VIIA and VIIB): The diagnostic value of collagen fibril ultrastructure. Am. J. Med. Genet. Part A.

[20] Ma H.P., Chang H.L., Bamodu O.A., Yadav V.K., Huang T.Y., Wu A.T.H., Yeh C.T., Tsai S.H., Lee W.H. (2019). Collagen1A1 (COL1A1) is a reliable biomarker and putative therapeutic target for hepatocellular carcinogenesis and metastasis. Cancers.

[21] Zang S., Guo R., Xing R., Zhang L., Li W., Zhao M., Fang J., Hu F., Kang B., Ren Y. (2014). Identification of differentially-expressed genes in intestinal gastric cancer by microarray analysis. Genom. Proteom. Bioinform..

[22] Shintani Y., Hollingsworth M.A., Wheelock M.J., Johnson K.R. (2006). Collagen I promotes metastasis in pancreatic cancer by activating c-Jun NH(2)-terminal kinase 1 and up-regulating N-cadherin expression. Cancer Res..

[23] Erceylan Ö.F., Savaş A., Göv E. (2021). Targeting the tumor stroma: Integrative analysis reveal GATA2 and TORYAIP1 as novel prognostic targets in breast and ovarian cancer. Turk. J. Biol..

[24] Sadelain M., Papapetrou E.P., Bushman F.D. (2011). Safe harbours for the integration of new DNA in the human genome. Nat. Rev. Cancer.

[25] Inukai S., Kock K.H., Bulyk M.L. (2017). Transcription factor-DNA binding: Beyond binding site motifs. Curr. Opin. Genet. Dev..

[26] Lu Q. (2005). Seamless cloning and gene fusion. Trends Biotechnol..

[27] Qi Z., Wang J., Li Y., Xu Y. (2022). MZF1 Transcriptionally activated microrna-328-3p suppresses the malignancy of stomach adenocarcinoma via inhibiting CD44. J. Immunol. Res..

[28] Liu T., Ye P., Ye Y., Lu S., Han B. (2020). Circular RNA hsa_circRNA_002178 silencing retards breast cancer progression via microRNA-328-3p-mediated inhibition of COL1A1. J. Cell Mol. Med..

[29] Kanki Y., Kohro T., Jiang S., Tsutsumi S., Mimura I., Suehiro J., Wada Y., Ohta Y., Ihara S., Iwanari H. (2011). Epigenetically coordinated GATA2 binding is necessary for endothelium-specific endomucin expression. EMBO J..

[30] Li L., Artlett C.M., Jimenez S.A., Hall D.J., Varga J. (1995). Positive regulation of human alpha 1 (I) collagen promoter activity by transcription factor Sp1. Gene.

[31] Nehls M.C., Rippe R.A., Veloz L., Brenner D.A. (1991). Transcription factors nuclear factor I and Sp1 interact with the murine collagen alpha 1 (I) promoter. Mol. Cell Biol..

[32] Chen L., Shioda T., Coser K.R., Lynch M.C., Yang C., Schmidt E.V. (2010). Genome-wide analysis of YY2 versus YY1 target genes. Nucleic Acids. Res..

[33] Martínez-Zamudio R.I., Roux P.F., de Freitas J., Robinson L., Doré G., Sun B., Belenki D., Milanovic M., Herbig U., Schmitt C.A. (2020). AP-1 imprints a reversible transcriptional programme of senescent cells. Nat. Cell Biol..

[34] Care M.A., Cocco M., Laye J.P., Barnes N., Huang Y., Wang M., Barrans S., Du M., Jack A., Westhead D.R. (2014). SPIB and BATF provide alternate determinants of IRF4 occupancy in diffuse large B-cell lymphoma linked to disease heterogeneity. Nucleic Acids. Res..

[35] Palomero J., Vegliante M.C., Rodríguez M.L., Eguileor A., Castellano G., Planas-Rigol E., Jares P., Ribera-Cortada I., Cid M.C., Campo E. (2014). SOX11 promotes tumor angiogenesis through transcriptional regulation of PDGFA in mantle cell lymphoma. Blood.

[36] Fang X., Yoon J.G., Li L., Yu W., Shao J., Hua D., Zheng S., Hood L., Goodlett D.R., Foltz G. (2011). The SOX2 response program in glioblastoma multiforme: An integrated ChIP-seq, expression microarray, and microRNA analysis. BMC Genom..

[37] ENCODE Project Consortium (2012). An integrated encyclopedia of DNA elements in the human genome. Nature.

[38] Sen G.L., Boxer L.D., Webster D.E., Bussat R.T., Qu K., Zarnegar B.J., Johnston D., Siprashvili Z., Khavari P.A. (2012). ZNF750 is a p63 target gene that induces KLF4 to drive terminal epidermal differentiation. Dev. Cell.

[39] Liska D.J., Reed M.J., Sage E.H., Bornstein P. (1994). Cell-specific expression of alpha 1(I) collagen-hGH minigenes in transgenic mice. J. Cell Biol..

[40] Jimenez S.A., Varga J., Olsen A., Li L., Diaz A., Herhal J., Koch J. (1994). Functional analysis of human alpha 1(I) procollagen gene promoter. Differential activity in collagen-producing and -nonproducing cells and response to transforming growth factor beta 1. J. Biol. Chem..

[41] Haberle V., Lenhard B. (2016). Promoter architectures and developmental gene regulation. Semin. Cell Dev. Biol..

[42] Annicotte J.S., Schoonjans K., Haby C., Auwerx J. (2001). An E-box in pGL3 reporter vectors precludes their use for the study of sterol regulatory element-binding proteins. Biotechniques.

[43] Wang J.M., Lang B., Zhu H.Y., Du H.T., Tian Y.M., Su Y.H. (2014). Cloning and transcriptional activity analysis of the porcine cofilin 2 gene promoter. Gene.

[44] Rippe R.A., Lorenzen S.I., Brenner D.A., Breindl M. (1989). Regulatory elements in the 5’-flanking region and the first intron contribute to transcriptional control of the mouse alpha 1 type I collagen gene. Mol. Cell Biol..

[45] Perrotti D., Melotti P., Skorski T., Casella I., Peschle C., Calabretta B. (1995). Overexpression of the zinc finger protein MZF1 inhibits hematopoietic development from embryonic stem cells: Correlation with negative regulation of CD34 and c-myb promoter activity. Mol. Cell Biol..

[46] Li J., Liao T., Liu H., Yuan H., Ouyang T., Wang J., Chai S., Li J., Chen J., Li X. (2021). Hypoxic glioma stem cell-derived exosomes containing linc01060 promote progression of glioma by regulating the MZF1/c-Myc/HIF1α axis. Cancer Res..

[47] Black A.R., Black J.D., Azizkhan-Clifford J. (2001). Sp1 and krüppel-like factor family of transcription factors in cell growth regulation and cancer. J. Cell Physiol..

[48] Gidoni D., Dynan W.S., Tjian R. (1984). Multiple specific contacts between a mammalian transcription factor and its cognate promoters. Nature.

[49] Jones K.A., Kadonaga J.T., Rosenfeld P.J., Kelly T.J., Tjian R. (1987). A cellular DNA-binding protein that activates eukaryotic transcription and DNA replication. Cell.

[50] O’Carroll A.M., Lolait S.J., Howell G.M. (2006). Transcriptional regulation of the rat apelin receptor gene: Promoter cloning and identification of an Sp1 site necessary for promoter activity. J. Mol. Endocrinol..

[51] Söding J., Lupas A.N. (2003). More than the sum of their parts: On the evolution of proteins from peptides. Bioessays.

[52] Bork P. (1991). Shuffled domains in extracellular proteins. FEBS Lett..

[53] Hunt L.T., Barker W.C. (1987). von Willebrand factor shares a distinctive cysteine-rich domain with thrombospondin and procollagen. Biochem. Biophys. Res. Commun..

[54] Reid K.B. (1993). Structure/function relationships in the collectins (mammalian lectins containing collagen-like regions). Biochem. Soc. Trans..

[55] Peterkofsky B. (1991). Ascorbate requirement for hydroxylation and secretion of procollagen: Relationship to inhibition of collagen synthesis in scurvy. Am. J. Clin. Nutr..

[56] McElroy K., Mouton L., Du Pasquier L., Qi W., Ebert D. (2011). Characterisation of a large family of polymorphic collagen-like proteins in the endospore-forming bacterium Pasteuria ramosa. Res. Microbiol..

