# Supplementary figures and images for: Molecular Characteristics and Promoter Analysis of Porcine COL1A1
Source: Genes (Basel). 2022 Oct 28;13(11):1971. doi: 10.3390/genes13111971 (PMC9689670; doi:10.3390/genes13111971)

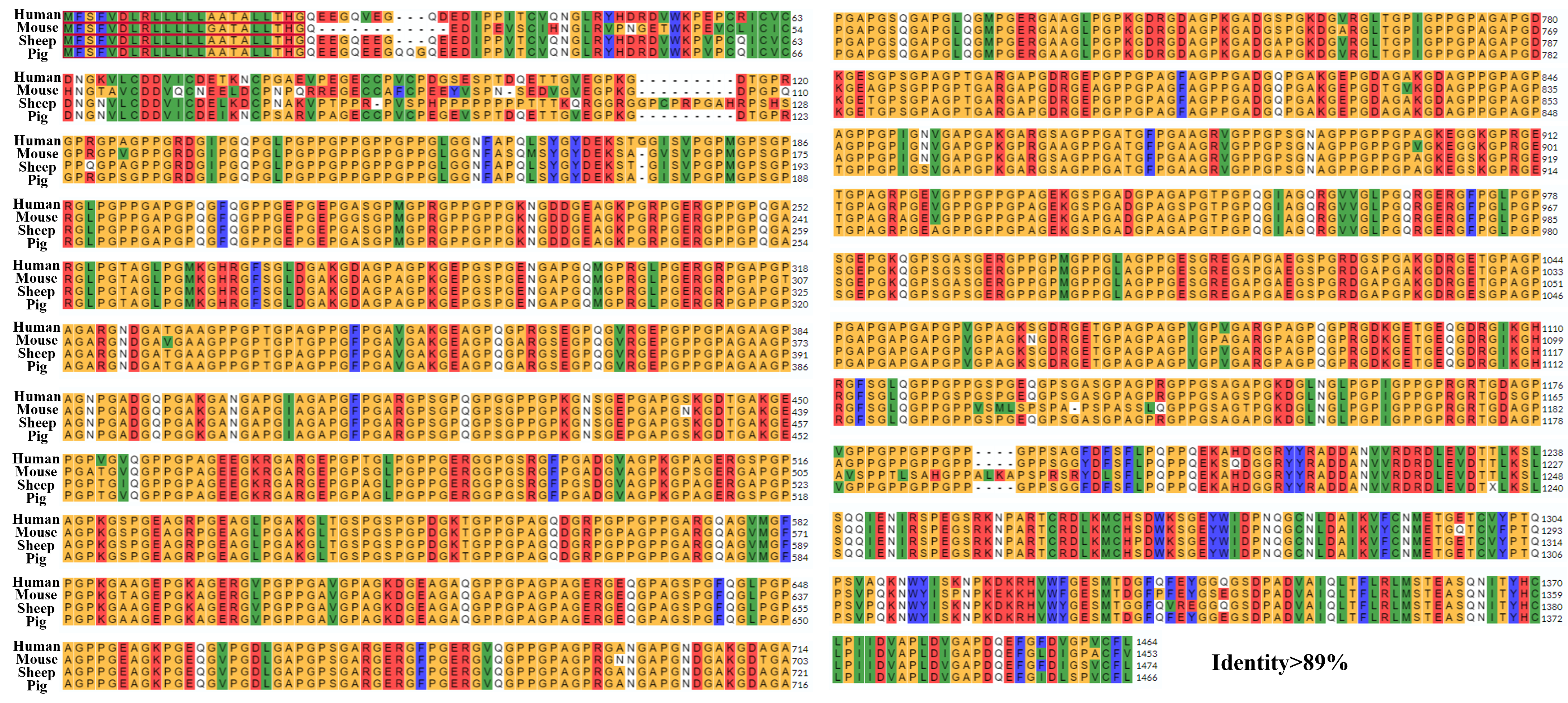

Supplement: Supplementary file 1 [file genes-13-01971-s001.zip › Supplementary Figure S1.tif]
